# Trichostatin C Synergistically Interacts with DNMT Inhibitor to Induce Antineoplastic Effect via Inhibition of Axl in Bladder and Lung Cancer Cells

**DOI:** 10.3390/ph17040425

**Published:** 2024-03-27

**Authors:** Chenyin Wang, Lijuan Lei, Yang Xu, Yan Li, Jing Zhang, Yanni Xu, Shuyi Si

**Affiliations:** NHC Key Laboratory of Biotechnology of Antibiotics, State Key Laboratory of Bioactive Substance and Function of Natural Medicines, National Center for New Microbial Drug Screening, Institute of Medicinal Biotechnology, Chinese Academy of Medical Sciences & Peking Union Medical College (CAMS & PUMC), Beijing 100050, China; wangchenyin@imb.pumc.edu.cn (C.W.); leilijuanlj@163.com (L.L.);

**Keywords:** histone deacetylase inhibitor, cancer, drug combination study, Axl, natural product, DNA methyltransferase inhibitor

## Abstract

Aberrant epigenetic modifications are fundamental contributors to the pathogenesis of various cancers. Consequently, targeting these aberrations with small molecules, such as histone deacetylase (HDAC) inhibitors and DNA methyltransferase (DNMT) inhibitors, presents a viable strategy for cancer therapy. The objective of this study is to assess the anti-cancer efficacy of trichostatin C (TSC), an analogue of trichostatin A sourced from the fermentation of *Streptomyces* sp. CPCC 203909. Our investigations reveal that TSC demonstrates potent activity against both human lung cancer and urothelial bladder cancer cell lines, with IC_50_ values in the low micromolar range. Moreover, TSC induces apoptosis mediated by caspase 3/7 and arrests the cell cycle at the G2/M phase. When combined with the DNMT inhibitor decitabine, TSC exhibits a synergistic anti-cancer effect. Additionally, protein analysis elucidates a significant reduction in the expression of the tyrosine kinase receptor Axl. Notably, elevated concentrations of TSC correlate with the up-regulation of the transcription factor forkhead box class O1 (FoxO1) and increased levels of the proapoptotic proteins Bim and p21. In conclusion, our findings suggest TSC as a promising anti-cancer agent with HDAC inhibitory activity. Furthermore, our results highlight the potential utility of TSC in combination with DNMT inhibitors for cancer treatment.

## 1. Introduction

Hyperactivation of histone deacetylase (HDAC; EC 3.5.1.98) has been correlated with initiation and progression in a number of malignancies [1]. HDACs are a group of enzymes used to remove acetyl groups from the lysine residues of histone to maintain a compacted chromatin structure, which leads to transcriptional repression and silencing of tumor suppressor genes [2]. To date, 11 HDACs have been identified and grouped into four subtypes, including class I, class IIa (HDAC4, 5, 7, and 9), class IIb (HDAC6 and 10), and class IV (HDAC11) depending on sequence homology to yeast. Class I HDACs, comprising HDAC1, 2, 3, and 8, have been found mainly in the nucleus and play a key role in cell survival. Class II HDACs, comprising class IIa (HDAC4, 5, 7, 9) and class IIb (HDAC 6 and 10), have tissue-specific roles, such as chondrocyte differentiation and maintaining vascular integrity [3]. Increased activity of HDACs, particularly class I HDACs, causes high cell proliferation rates which are reflected in the cell cycle progression and low apoptosis [4]. Therefore, developing a therapeutical approach against cancer by targeting HDAC is reasonable.

Since the first HDAC inhibitor, vorinostat, approved for the treatment of cutaneous T-cell lymphoma in 2006, five HDAC inhibitors have been approved for T-cell lymphoma and solid tumors [5]. According to its target, HDAC inhibitors are classified into pan-HDAC inhibitors and selective HDAC inhibitors. The limitation of pan-HDAC inhibitors has an off-target effect due to the broad targets affected [6]. Based on chemical structures, HDAC inhibitors are grouped into three families: hydroxamic acids, benzamides, and cyclic tetrapeptides [7]. Trichostatin A, a typical hydroxamic acid with a more rigid alkenyl group, has inspired the design and optimization of many novel HDAC inhibitors such as Belinostat (approved in 2014 for peripheral T-cell lymphoma). Mechanism studies of trichostatin A suggest a critical role of hydroxamic acid as a zinc chelator, which blocks the catalytic activity of HDAC [8]. The first isolation of trichostatin A was reported by Tsuji and colleagues from the metabolites of *Streptomyces hygroscopicus* in 1976 [9]. Then, trichostatin A was intensively studied, and multiple bioactivities have been reported, including anti-diabetic, anti-inflammatory, and antioxidant activities [8].

Receptor tyrosine kinases (RTKs) are a family of transmembrane proteins mediating signaling transduction for cell-to-cell communication [10,11]. A total of 58 RTKs have been found in humans, and many of them play an essential role in regulating differentiation, cell growth, and cell metabolism [12]. Since over-expressed and over-activated RTKs occur frequently in various cancer cells and thus result in dysregulated proliferation, RTKs therefore provide a selective therapeutic target to combat cancer [13]. Beyond the extensively studied RTKs such as epidermal growth factor receptor (EGFR) and vascular endothelial growth factor receptor (VEGFR), Axl has emerged as a promising target due to its role in cancer cell proliferation, migration, and invasion [14]. Interestingly, recent studies have demonstrated the regulation of RTKs by epigenetic mechanisms such as HDAC inhibition [15]. In colorectal cancer, for instance, the expression of EGFR decreased as a result of histone hypoacetylation, and the dissociation of SP1, HDAC3, and CBP induced transcriptional repression [16]. In addition, a study focused on cancer stem cells revealed that HDAC promotes the formation of the drug-tolerant persister (DTP) phenotype, which is required for the activation of IGF-1R signaling [17]. Taken together, these data suggest a mechanism-linked correlation between HDAC activity and the activation of RTKs. 

Based on the solid evidence that targeting HDAC is an effective approach to combat cancer, in current study, the anti-cancer effect of trichostatin C (TSC), an analogue of trichostatin A isolated from fermentation of *Streptomyces* sp. CPCC 203909, was investigated.

## 2. Results

### 2.1. Isolation and Identification of TSC

The isolation of TSC was conducted similarly to what was previously described [18]. The compound was isolated as a white amorphous powder and identified as TSC based on its identical ESI-MS, ^1^H and ^13^C NMR data to those published in the literature [19]. ESI-MS: *m*/*z* 465.2 [M + H]^+^; ^1^H NMR (600 MHz, CD_3_OD) δ_H_: 7.86 (2H, d, *J* = 9.2 Hz, H-9, 13), 7.27 (1H, d, *J* = 15.6 Hz, H-3), 6.72 (2H, d, *J* = 9.2 Hz, H-10, 12), 5.97 (1H, d, *J* = 9.6 Hz, H-5), 5.87 (1H, d, *J* = 15.6 Hz, H-2), 4.54 (1H, dq, *J* = 9.6, 6.0 Hz, H-6), 3.91 (1H, dd, *J* = 12.0, 2.4 Hz, H-6′a), 3.67 (1H, dd, *J* = 12.0, 6.0 Hz, H-6′b), 3.40 (1H, t, *J* = 9.0 Hz, H-3′), 3.34 (1H, m, H-5′), 3.32 (1H, m, H-2′), 3.28 (1H, m, H-4′), 3.06 (6H, s, N-Me_2_), 1.93 (3H, s, 4-Me), 1.27 (3H, d, *J* = 6.0 Hz, 6-Me); ^13^C NMR (600 MHz, CD_3_OD) δ_C_: 167.1 (C-1), 116.3 (C-2), 147.8 (C-3), 134.4 (C-4), 142.7 (C-5), 41.8 (C-6), 201.4 (C-7), 124.8 (C-8), 132.1 (C-9, 13), 112.1 (C-10, 12), 155.6 (C-11), 40.2 (N-Me_2_), 12.9 (4-Me), 18.4 (6-Me), 107.8 (C-1′), 73.2 (C-2′), 77.6 (C-3′), 71.4 (C-4′), 78.5 (C-5′), 62.9 (C-6′).

### 2.2. TSC Exhibits Anti-Proliferation Effect in Cancer Cell Line with HDAC Inhibitory Activity

**Figure 1:**
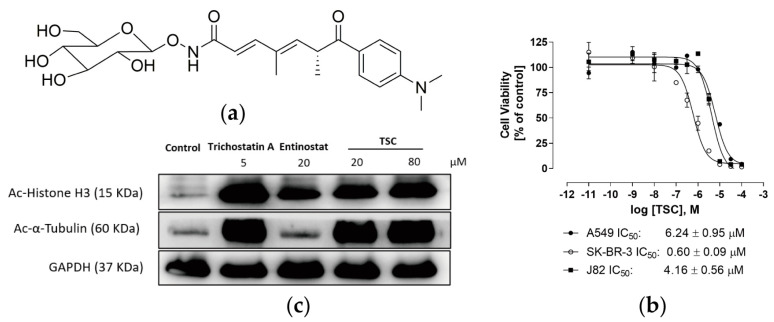
Characterization of TSC of its cytotoxicity and HDAC inhibitory activity. Structure of TSC (TSC) isolated from fermentation of *Streptomyces* sp. CPCC 203,909 (**a**). Concentration–effect curves of TSC in A549, SK-BR-3, and J82 (**b**). Acetylation levels of α-Tubulin and Histone H3. J82 cells were treated with the indicated concentrations of trichostatin A, Entinostat, and TSC (**c**).

The cytotoxicity of TSC Figure 1a was tested using a 3-(4,5-dimethylthiazol-2-yl)-2,5-diphenyltetrazolium bromide (MTT) assay in three cancer cell lines including SK-BR-3 (human breast cancer cell line), A549 (human lung cancer cell line), and J82 (human urothelial bladder cancer cell line). As shown in Figure 1b, the cell proliferation was inhibited significantly in a dose-dependent manner by TSC. Upon 72 h treatment, the IC_50_ values of TSC were 6.24 μM and 4.16 μM in the A549 and J82 cell lines, respectively. In the breast cancer cell line SK-BR-3, TSC showed potent activity with an IC_50_ value of 0.60 μM. The result of cell viability assay of trichostatin A is shown in Figure S1. Since TSC is an analogue of trichostatin A, HDAC activity was tested after treatment with trichostatin A, TSC, and class I selective HDAC inhibitor entinostat by Western blot analysis. It is known that acetylation of lysine residue on histone H3 and α-tubulin is an indicator of the inhibition of HDAC1 and HDAC6; moreover, the expression of ac-histone H3 and ac-α-tubulin was evaluated after 24 h drug treatment. A substantial increased expression of ac-histone H3 was observed with TSC treatment, indicating the increased level of acetylation of histone H3 induced by the decreased activity of HDAC1 Figure 1c. In addition, as the marker of suppressed HDAC6, the protein level of ac-α-tubulin was up-regulated after TSC treatment. As a non-selective HDAC inhibitor, trichostatin A inhibited activity of both HDAC1 and HDAC6. In contrast to TSC and trichosctatin A, the class I selective HDAC inhibitor entinostat only inhibited the activity of HDAC1, which is in agreement with its HDAC inhibition profile [20].

### 2.3. TSC Induces Apoptosis by the Activation of Caspase 3/7 Cancer Cell Line

**Figure 2:**
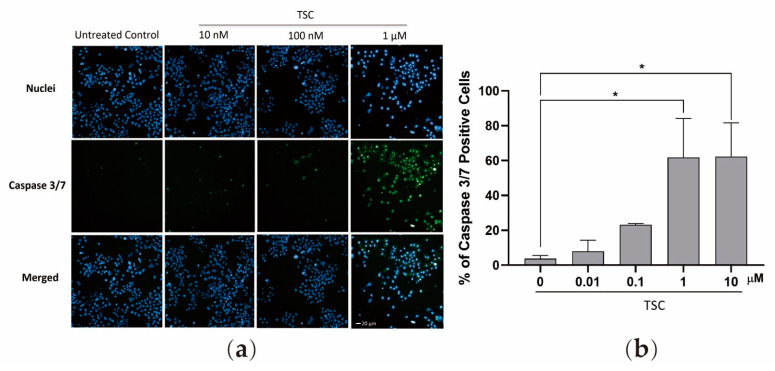
TSC-induced activation of caspase 3/7. J82 cells were treated with TSC with TSC for 48 h and labeled with Hoechst 33,342 for cell nucleus and CellEvent caspase 3/7 reagent for activated caspase 3/7 (**a**). Percentage of caspase 3/7-positive cells (**b**). The two-tailed Student’s *t*-test or ANOVA was applied. Results are presented as mean ± SEM from three experiments. * *p* < 0.05 compared to control cells.

Depending on their function in the process of apoptosis, caspases are divided into “initiator caspases” and “executioner caspases”. Caspase 3 and 7, belonging to the group of executioner caspase, share similar sequences with each other. The activation of caspase 3 and 7 is essential for the onset of apoptosis in the terminal phase [21]. Given the fact that TSC inhibited the proliferation of cancer cells and HDAC activity, caspase 3 and 7 activation assay was performed to determine the effect of TSC for the induction of caspase-mediated apoptosis. In untreated control cells, the activated caspase 3/7 was 3.8%. While 72 h incubation with TSC, the percentage of caspase 3/7-positive cells were increased to 23.1%, 61.7%, and 62.3% with 0.1 μM, 1 μM, and 10 μM TSC, respectively (Figure 2).

### 2.4. Combining TSC and Decitabine Leads to Synergistic Cytotoxicity in Cancer Cells

**Figure 3:**
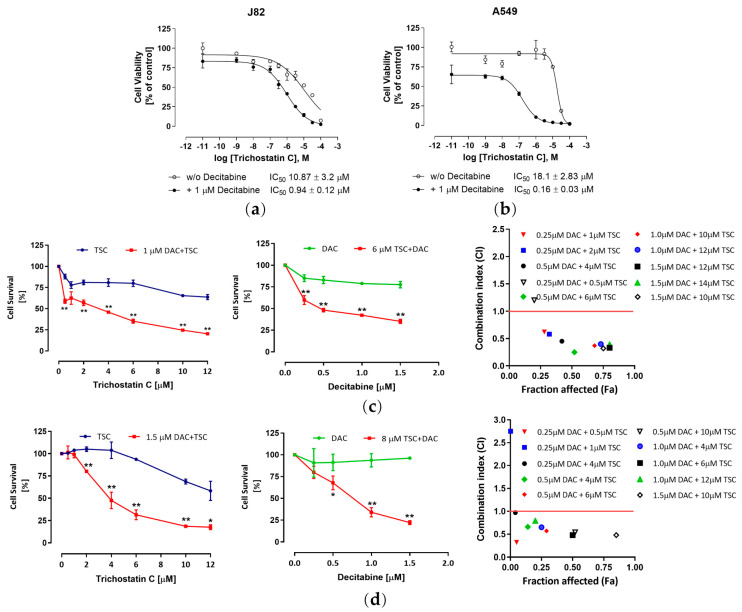
Synergistic anti-cancer effect of TSC and decitabine in human urothelial bladder cancer cell line J82 and human lung cancer cell line A549. Total of 48 h incubation with decitabine prior to TSC significantly increased cytotoxicity of TSC in J82 (**a**) and in A549 (**b**) cell lines. Synergistic effect was determined by the Chou–Talalay method in J82 (**c**) and A549 (**d**) cell lines. Left panel and middle panels: cell survival rate upon treatment with TSC and decitabine alone or in combination. The right panel indicates the fraction of cells affected (Fa) and CI. Synergistic effect is defined by CI < 1. Results are presented as mean ± SD from three experiments. ** p < 0.01, * p < 0.05, combination treatment vs. single treatment.

The data shown above demonstrate the inhibitory activity of HDAC and activation of caspase 3/7 by TSC. Because epigenetic events such as histone deacetylation and DNA methylation work to accompany each other in regulating chromatin structure and gene expression, it is thus reasonable to conduct a synergism study of TSC and DNA methyltransferase (DNMT) inhibitors. As expected, combining the DNMT inhibitor decitabine enhanced the activity of TSC significantly. Figure 3a,b show the dose-dependent curves of TSC single treatment and in combination with decitabine. In the A549 cell line, the IC_50_ value of TSC decreased from 18.1 μM to 0.16 μM when combining with decitabine. In J82 cells, the shift factor (the ratio between the IC_50_ value of single treatment and combination treatment) was 11.6, corresponding to the IC_50_ value of 10.9 μM and 0.94 μM with TSC treated alone and combination, respectively. The synergistic effect of TSC and decitabine was then proved by the Chou–Talalay method. According to this model, the synergistic effect is confirmed if the drug combination index (CI) is below 1 [22]. In this experiment, various concentrations of TSC and decitabine were tested alone and in combination. Strong synergistic cytotoxicity was observed in both A549 and J82 cells (Figure 3c,d). Additionally, a significant decrease in cell survival was seen in the combination treatment compared with either TSC or decitabine alone. 

### 2.5. TSC and Decitabine Synergistically Induces Apoptosis

**Figure 4:**
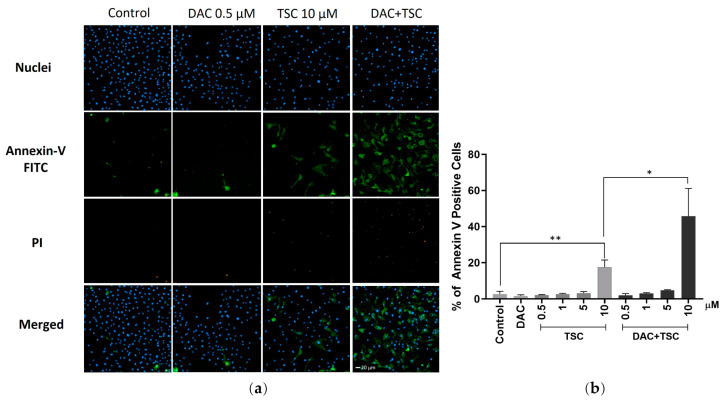
TSC and decitabine synergistically induces apoptosis. Cells were treated with decitabine for 48 h followed by another 48 h treatment with TSC. After drug treatment, cells were labeled with Hoechst 33342, Annexin-V FITC, and PI (**a**). Quantification of apoptotic cells are displayed (**b**). Either the two-tailed Student’s *t*-test or ANOVA was applied. Results are presented as mean ± SEM from three experiments. ** *p* < 0.01, * *p* < 0.05 as compared with control cells.

The synergism of TSC and decitabine was then determined via an apoptosis assay by using fluorescence-based high-content analysis. The J82 cells were exposed to the two agents separately and in combination, and then the cells were labelled with Annexin V and propidium iodide for apoptosis analysis. Treatment with TSC and decitabine alone only induced a moderate level of apoptotic cells. When a combination of 10 μM TSC and 1 μM decitabine was applied, however, 46% of cells were detected with Annexin V, which is only detectable when it binds to phosphatidylserine undergoing apoptosis (Figure 4b). These results are in well agreement with the synergistic study described above.

### 2.6. TSC Induces G2/M Phase Cell Cycle Arrest in Bladder Cancer Cells 

**Figure 5:**
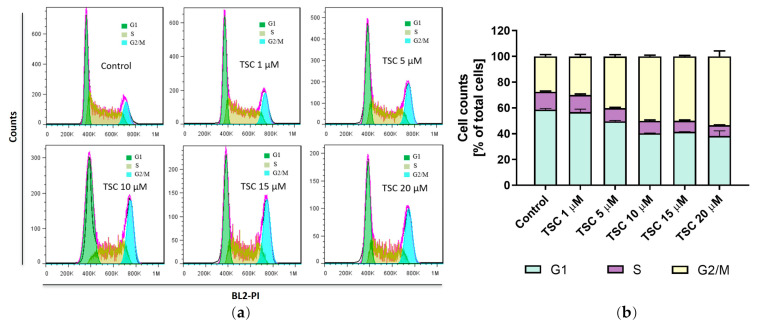
TSC leads to G2/M phase cell cycle arrest. Cells were incubated with TSC for 48 h. The region of corresponding cell cycle phase was defined and calculated (**a**). Quantification of the cell cycle distribution after treatment of TSC (**b**). Results are presented as the mean ± SD of three independent experiments.

To test whether TSC affects cell cycle distribution, the cells treated with TSC were analyzed by flow cytometry with PI staining. In the untreated group, the cell population in the G1, S, and G2/M phase was 58.73%, 13.85%, and 27.43%, respectively. Treatment with TSC induced G2/M phase arrest in a dose-dependent manner. The number of cells in the G2/M phase increased from 29.98% to 40.13% and 50.1% with 1 μM, 5 μM, and 10 μM treatment with TSC, respectively (Figure 5). These data thus indicate that TSC induced inhibition of cell proliferation, which was not only achieved by the caspase 3/7-mediated apoptosis, but also associated with cell cycle arrest in the G2/M cell phase. 

### 2.7. TSC Alters Expression of Axl and FoxO1 Pathway 

**Figure 6:**
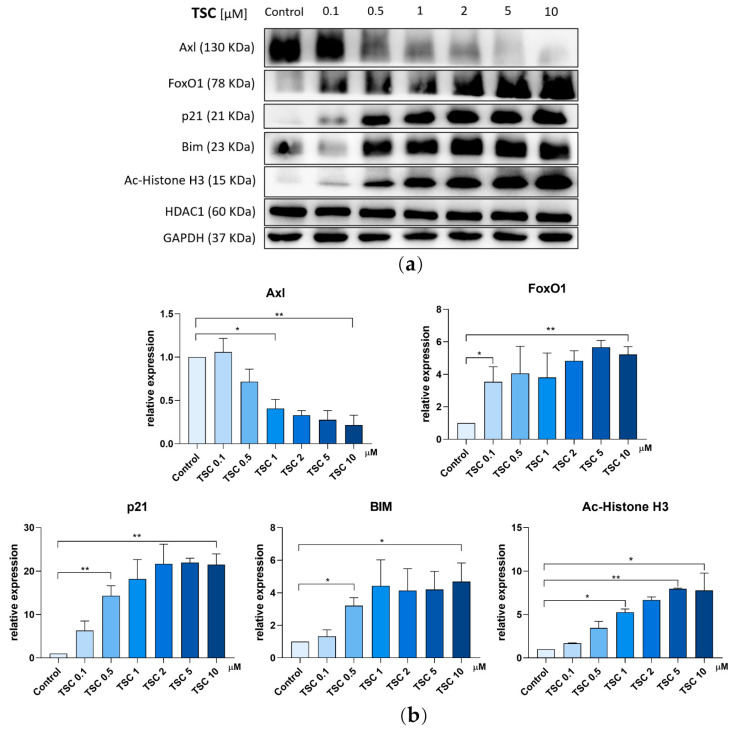
TSC altered expression of Axl and targets involved in FoxO1 pathway. Representative Western blot analysis of J82 cells upon the treatment of TSC for 48 h (**a**). Densitometrically measure expression of proteins relative to GAPDH are shown with three independent experiments (**b**). Results shown are the mean ± SD from three independent experiments. ** *p* < 0.01, * *p* < 0.05 as compared with control cells.

In order to study the signaling pathways are potentially affected by TSC, Western blot was performed after the J82 cells were treated with TSC. First, as a member of the RTK family, the expression of Axl was down-regulated with the increased concentration of TSC. A total of 10 μM of TSC led to a significant decrease in Axl expression. Second, the transcriptional factor Forkhead box O 1 (FoxO1) was enhanced upon the treatment of TSC. Then, a set of downstream targets of FoxO1 including p21 and Bim were evaluated. As shown in Figure 6, p21, the key regulator of the cell cycle, was up-regulated with increased concentrations of TSC. Another downstream target, Bim, the pro-apoptotic protein, was also increased following the treatment of TSC. Together, these data are consistent with the results shown above, which indicate that the anti-proliferation effects of TSC are associated with apoptosis induction and cell cycle arrest. 

## 3. Discussion

HDAC-mediated removal of the acetyl group of lysine residue on histone has been widely reported for its function in carcinogenesis. A number of HDAC inhibitors have received approval mainly for the treatment of lymphoma, and their anti-cancer effects have been validated clinically. In this study, we described an analogue of trichstatin A, isolated from the fermentation of *Streptomyces* sp. CPCC 203909, showing anti-proliferation effects in lung cancer and urothelial bladder cancer cell lines via the inhibition of HDAC activity. Since histone deacetylation cooperates with DNA methylation to regulate chromatin structure and gene expression, our study also tested the synergism effect of TSC and DNMT inhibitors. 

As an analogue of trichostatin A, the HDAC inhibition activity of TSC was depicted. Our study suggests that both HDAC1 and HDAC6 were inhibited by TSC to a similar extent as trichostatin A. Since class I HDAC has a pivotal role in cell survival and proliferation, it is believed that hyperactivation of HDAC1 is positively correlated with cancer development [23]. Unlike HDAC1, which is located in the nucleus for its function in regulating gene transcription, HDAC6 is mainly located in the cytoplasm [24]. Among 11 zinc-dependent deacetylases, HDAC6 is a unique HDAC which exclusively deacetylates several proteins including heat shock protein 90 (HSP90), α-tubulin, and chaperone proteins [25]. Studies have revealed that inhibition of HDAC6 results in apoptosis induction, which is in agreement with our result showing the reduced activity of HDAC6 by TSC and apoptosis induction in urothelial bladder cancer cell line J82. 

Our study demonstrates that TSC and decitabine synergistically affect the viability of lung cancer cell line A549 and urothelial bladder cancer cell line J82 by caspase 3/7-mediated apoptosis. Importantly, both decitabine and TSC alone show a moderate level of caspase 3/7 activation. However, when the two agents were combined, a significant increase in caspase 3/7-positive cells was observed. Furthermore, regarding the anti-cancer mechanisms of TSC, we found out that Axl was substantially down-regulated upon the treatment of TSC. Axl, belonging to the family of TAM (Tyro, Axl, Mer) receptor tyrosine kinase, has attracted great attention due its intensive involvement in cancer metastasis, progression, and therapeutic resistance [26,27]. Conversely, a reduced level of Axl expression is associated with good prognosis and longer overall survival [28]. Therefore, the reduced expression of Axl found in our study partially explained the cytotoxicity induced by TSC. The inhibition of Axl further affected the cell signaling transduction involved in cell proliferation. For instance, the expression of transcription factor FoxO1 was increased following the activation of its downstream targets such as proapoptotic protein Bim and cell cycle inhibitor p21. The increased expression of Bim and p21 contributed to the induction of apoptosis and cell cycle arrest.

In summary, our present results suggest the synergistic anti-cancer effects of TSC when combining the DNMT inhibitor decitabine in bladder and lung cancer cells. Our initial data provide a highly potential drug combination strategy for the treatment of bladder and lung cancer. Further studies, including in vivo and pharmacokinetic studies, are needed for laying the groundwork of TSC in cancer treatment.

## 4. Materials and Methods

### 4.1. Materials

Dulbecco’s Modified Eagle Medium (DMEM) was ordered from Gibco, Thermo Fisher Scientific (Waltham, MA, USA), while F-12K medium and McCoy’s 5A was obtained from the American Type Culture Collection (ATCC, Manassas, VA, USA). Penicillin/streptomycin (pen/strep) solution [10,000 U/mL; 10 mg/mL] and fetal bovine serum were obtained from HyClone Laboratories (Logan, UT, USA). Trypsin-EDTA was purchased from Cytiva (Marlborough, MA, USA). Decitabine (Selleckchem, Houston, TX, USA) was prepared as a 10 mM stock solution in dimethyl sulfoxide (DMSO). Hoechst 33,342 stain was procured from Solarbio (Beijing, China), while the CellEvent Caspase-3/7 green detection reagent was obtained from Invitrogen (Carlsbad, CA, USA). 3-(4,5-dimethylthiazol-2-yl)-2,5-diphenyltetrazolium bromide (MTT) was sourced from Solarbio and dissolved in phosphate-buffered saline (PBS) to achieve a concentration of 5 mg/mL. For Western blotting, HRP-conjugated secondary antibodies were obtained from BioRad (Hercules, CA, USA).

### 4.2. Fermentation of Streptomyces sp. CPCC 203909 and Isolation of TSC

*Streptomyces* sp. CPCC203909, deposited in the China Pharmaceutical Culture Collection (Institute of Medicinal Biotechnology, Chinese Academy of Medical Sciences (CAMS) and Peking Union Medical College (PUMC)), was cultivated on BD Difco ISP Medium slants at 28 °C. Subsequently, the strain was inoculated into 3 mL of A2 liquid medium comprising 1.0% glucose, 3.0% amylogen, 2.0% cottonseed meal, 0.3% yeast extract, 0.3% ammonium sulfate, 0.1% magnesium sulfate, 0.1% dipotassium hydrogen phosphate, 0.1% sodium chloride, and 0.5% calcium carbonate. The inoculated media were incubated at 28 °C on a rotary shaker at 180 rpm for 2 days post sterilization. To establish the seed culture, the inoculum was transferred to 100 mL of A2 liquid medium and maintained on a rotary shaker at 28 °C and 180 rpm for 48 h. Subsequently, fermentation was conducted using solid rice medium following autoclaving. The seed culture was then added and incubated for 30 days at 28 °C.

Following the 30-day incubation period, the fermentation products were soaked in ethanol overnight, and the liquid was filtered and subjected to extraction with ethyl acetate (EtOAc). The organic solvent was evaporated using a rotary evaporator to obtain a crude extract (111.36 g).

The crude extract was fractionated via silica column chromatography using CH_2_Cl_2_-MeOH with varying ratios (50:1, 30:1, 10:1, 5:1) along with pure methanol. The methanol fraction yielded four sub-fractions, with fraction 1 (1.72 g) further separated using RP-18 chromatography with a mobile phase of 35% acetonitrile in distilled water at 5 mL/min, resulting in four additional sub-fractions. Subfraction 3 was subjected to purification by semipreparative RP high-performance liquid chromatography (HPLC; mobile phase: 30% acetonitrile in water containing 0.1% trifluoroacetic acid) to isolate TSC (8.62 mg).

### 4.3. Cell Culture

The human lung cancer cell line A549 was purchased from the American Type Culture Collection (ATCC, Manassas, VA, USA). The human urothelial bladder cancer cell line J82 was obtained from Merck Millipore (Burlington, MA, USA). The human breast cancer cell line SK-BR-3 was ordered from Procell (Wuhan, China). A549, J82, and SK-BR-3 cells were maintained F-12K medium, DMEM, and McCoy’s 5A, respectively. All of the cells were supplemented with 10% fetal bovine serum, 120 µg/mL streptomycin, and 120 U/mL penicillin. Cell cultures were incubated in a humidified atmosphere containing 5% CO_2_ at 37 °C. Upon reaching 80–90% confluence, cells were either passaged or harvested for the respective assays.

### 4.4. Cell Viability Assay

The MTT assay was performed to assess cell viability, following established protocols [29]. Briefly, cells were seeded into 96-well plates (Corning Inc., Corning, NY, USA) one day prior to drug treatment. Subsequently, the cells were exposed to varying concentrations of TSC and decitabine. Following the incubation period with the drugs, 25 µL of MTT solution (5 mg/mL) was added to each well and allowed to incubate for 15 min. The medium and MTT solution mixture was then aspirated, and 50 µL of DMSO was added to dissolve the formazan crystals. The absorbance of each well was measured at 544 nm (test wavelength) and 690 nm (background) using the MultiskanTM FC Microplate Photometer (Thermo Fisher Scientific, Waltham, MA, USA). The IC_50_ values, the concentration of the cytotoxic agent that led to a decrease of 50% in the recorded signal, were calculated by using GraphPad Prism 8.0 (GraphPad Software Inc., San Diego, CA, USA) [29].

### 4.5. Synergistic Study

To assess the synergistic effects of TSC and decitabine, we evaluated the rates of cell growth inhibition for each agent using the MTT assay. We calculated combination indexes (CIs) utilizing Compusyn software 1.0 (ComboSyn, Inc., Paramus, NJ, USA), applying the Chou–Talalay method. Synergism, additive effects, and antagonism were defined by CI values < 1, =1, and >1, respectively [30]. 

### 4.6. Apoptosis Assay

The apoptosis assay was conducted using high-content screening. Cells were seeded on 96-well plates and incubated overnight. After incubation with TSC and DAC, the apoptotic cells was evaluated using Annexin V-FITC apoptosis Kit (Beyotime, Haimen, China) according to the manufacturer’s instructions. Briefly, the supernatant was removed and the mixture of fluorescent solution containing Annexin V-FITC, propidium iodide, and Hoechst 33,342 was added to the cells for 30 min at room temperature. The cells were then scanned by the high-content analysis system Operetta CLS (PerkinElmer, Waltham, MA, USA) using excitation filters at 530, 488, and 386 nm for propidium iodide, FITC, and Hoechst 33342, respectively. The acquired images were analyzed by Harmony 4.9 high-content imaging and analysis software. 

### 4.7. Cell Cycle Analysis

For cell cycle analysis, the cells were plated on a 6-well plate and incubated overnight. The cells were then treated with TSC for 48 h. After incubation, the cells were collected and washed with PBS and then fixed in 70% ice-cold ethanol at −20 °C for 24 h. The fixed cells were stained by using the Cell Cycle and Apoptosis Analysis Kit (Beyotime, China) following the manufacturer’s instruction after the cells were washed with cold PBS. The cells were stained for 30 min at 37 °C (protected from light). The DNA content of the cells was measured by flow cytometer (Attune NxT, Thermo Fisher Scientific, Waltham, MA, USA).

### 4.8. Caspase 3/7 Activation

Caspase 3/7 activity upon TSC treatment was determined using high-content analysis. The bladder cancer cell line J82 was subjected to the same treatment protocol as described for the apoptosis assay. After the incubation with TSC, the medium was aspirated, and the cells were stained with mixture of Hoechst 33342 and CellEvent^TM^ caspase-3/7 Green Detection Reagent for 30 min. Imaging was conducted using Operetta CLS (PerkinElmer, Waltham, MA) with excitation filters at 386 nm and 488 nm for Hoechst 33342 and the caspase 3/7 Green Detection Reagent, respectively. The proportion of activated caspase 3/7 cells was quantified using Harmony 4.9 high-content imaging and analysis software Harmony 4.9 (PerkinElmer, Waltham, MA, USA).

### 4.9. Western Blot Analysis

Total protein isolation and Western blot analysis were performed as previously described [31]. Briefly, treated cells were lysed with RIPA buffer (CWBIO, Taizhou, China) containing protease and phosphatase inhibitor (CWBIO, Taizhou, China) and boiled at 95 °C with 4X Laemmli Sample buffer (BioRad, Hercules, CA, USA) containing 10% β-mercaptoethanol for 5 min. The protein concentration was measured by Pierce BCA protein assay (Thermo Scientific, Rockford, IL, USA). The protein samples were loaded with equal amount onto gel for electrophoresis. Proteins were transferred onto the PVDF (polyvinylidene difluoride) membrane (Merck Millipore, Darmstadt, Germany) by 1 h blotting. Blots were then blocked in Tris Buffered Saline-0.1% Tween 20 (TBST) 5% milk solution or TBST-5% BSA (Lablead, Biotechnology, Beijing, China) for 1 h. Blots with primary antibodies were incubated at 4 °C overnight. Blots were then washed 3 times with TBST and followed with HRP-conjugated secondary antibody incubation for 1 h. Blots were then washed again 3 times with TBST. Images were obtained using the Immobilon Western Chemiluminescent HRP Substrate (Merck Millipore, Burlington, MA, USA) and the ChemiDoc MP (BioRad, Hercules, CA, USA). Densitometrically measurement and analysis of protein expression was performed by using Image J 1.52 software.

### 4.10. Statistical Analysis

Statistical analysis was conducted using GraphPad Prism 8.0 (GraphPad Software Inc., San Diego, CA, USA). Concentration–effect curves were generated through non-linear regression curve fitting utilizing the four-parameter logistic equation with a variable hill slope. All assays were performed in at least two independent experiments, each conducted in triplicate. A significance test was performed using either the two-tailed Student’s *t*-test or analysis of variance (ANOVA). A *p*-value < 0.05 was considered statistically significant. Data are presented as mean ± SEM.

## 5. Conclusions

In conclusion, our in vitro study elucidates that TSC exhibits its anti-cancer effects through HDAC inhibition. Furthermore, our findings highlight the pivotal role of TSC in downregulating Axl expression and activating FoxO1, influencing the fate of urothelial bladder cancer cells. Moreover, the synergistic anti-cancer effects observed with TSC and decitabine in both lung and bladder cancer cell lines reveal the potential therapeutic efficacy of this combination. Overall, our study provides compelling evidence that TSC, as an analogue of trichostatin A but with reduced toxicity, is a promising anti-cancer agent via HDAC inhibition with natural origin.

## Data Availability

Data is contained within the article and Supplementary Materials.

## Supplementary Materials

The following supporting information can be downloaded at: https://www.mdpi.com/article/10.3390/ph17040425/s1, Figure S1. Evaluation of cytotoxic effect of TSA. Figure S2. HPLC profile of cell lysate supernatant after TSA and TSC treatment.
