# Supplementary material for: Trichostatin C Synergistically Interacts with DNMT Inhibitor to Induce Antineoplastic Effect via Inhibition of Axl in Bladder and Lung Cancer Cells
Source: Pharmaceuticals (Basel). 2024 Mar 27;17(4):425. doi: 10.3390/ph17040425 (PMC11053535; doi:10.3390/ph17040425)
Supplement: Supplementary file 1 [file pharmaceuticals-17-00425-s001.zip › pharmaceuticals-2926430-supplementary.pdf]

Supplemental data

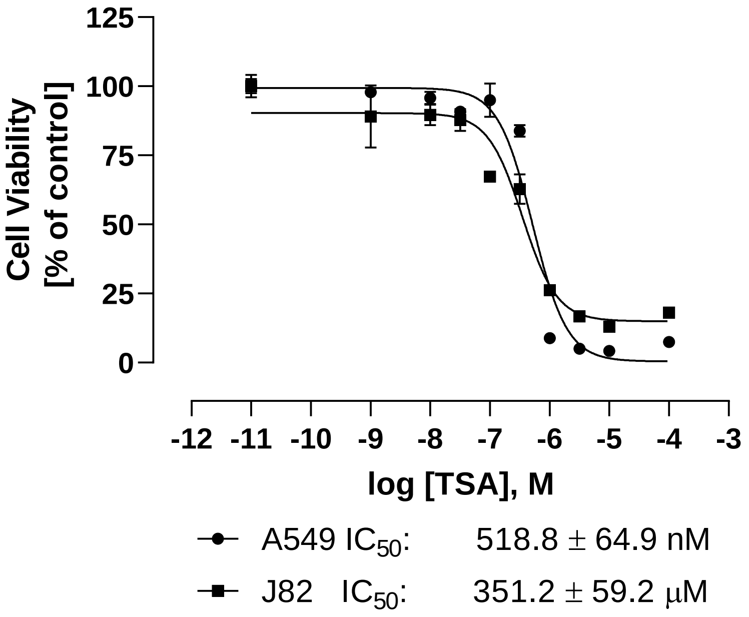

Figure S1. Evaluation of cytotoxic effect of TSA.

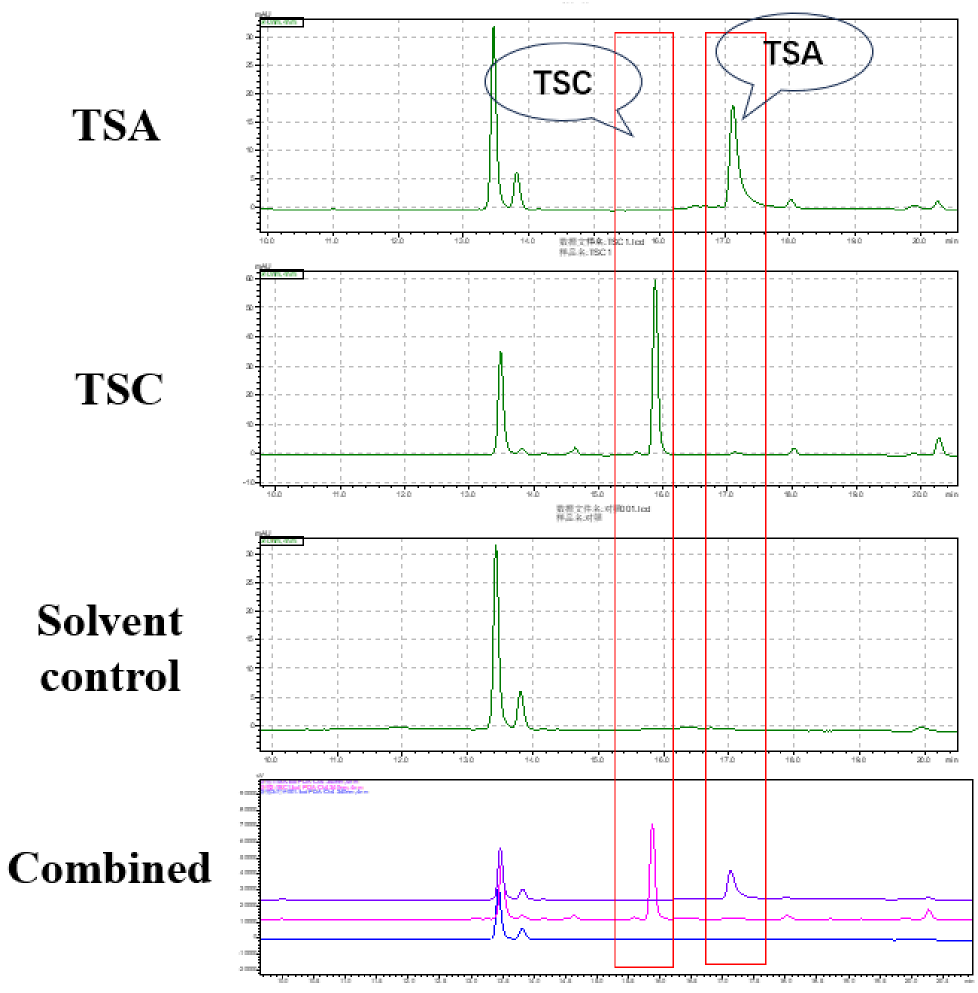

Figure S2. HPLC profile of cell lysate supernatant after TSA and TSC treatment
